# Gene-edited pigs: a translational model for human food allergy against alpha-Gal and anaphylaxis

**DOI:** 10.3389/fimmu.2024.1358178

**Published:** 2024-02-26

**Authors:** Ying Wang, Miriam Hils, Andrea Fischer, Florian Wölbing, Tilo Biedermann, Angelika Schnieke, Konrad Fischer

**Affiliations:** ^1^ Chair of Livestock Biotechnology, School of Life Sciences Weihenstephan, Technical University of Munich, Freising, Germany; ^2^ Department of Dermatology and Allergy Biederstein, School of Medicine, Technical University of Munich, Munich, Germany

**Keywords:** α-Gal allergy, anaphylaxis to α-Gal, intracutaneous sensitization, red meat allergy, translational pig model

## Abstract

The prevalence of food allergy is rising and is estimated to approach 10%. Red meat allergy is the first known food allergy elicited by immunoglobulin E (IgE) antibodies recognizing a carbohydrate. Due to the loss of function of the alpha−1,3−galactosyltransferase (*GGTA1)* gene in humans, the disaccharide galactose-α-1,3-galactose (α-Gal) cannot be synthesized and therefore became immunogenic. IgE sensitization is elicited through the skin by repetitive tick bites transmitting α-Gal. The underlying mechanisms regarding innate and adaptive immune cell activation, including the B-cell isotype switch to IgE, are poorly understood, requiring further research and physiologically relevant animal models. Here, we describe a new animal model of red meat allergy using percutaneous α-Gal sensitization of gene-edited *GGTA1*-deficient pigs. Total and α-Gal-specific IgG, IgG1, IgG2, IgG4, and IgE levels were tracked. Further key factors associated with allergic skin inflammation, type 2 immunity, and allergy development were measured in PBMCs and skin samples. Significant increases in α-Gal-specific IgG1 and IgE levels indicated successful sensitization to the allergen α-Gal. Intracutaneous sensitizations with α-Gal recruited lymphocytes to the skin, including elevated numbers of T helper 2 (Th2) cells. Finally, α-Gal-sensitized pigs not only recognized α-Gal as non-self-antigen following α-Gal exposure through the skin but also developed anaphylaxis upon antigen challenge. Based on the similarities between the porcine and human skin, this new large animal model for α-Gal allergy should help to unveil the consecutive steps of cutaneous sensitization and aid the development of prophylactic and treatment interventions.

## Introduction

Food allergies affect at least 3%–6% of the world population and can cause life-threatening symptoms (1). While the allergen itself is often known, the various steps of sensitization and the underlying immunopathogenic mechanisms, leading to severe allergic responses, remain largely unknown. We therefore established the pig as a new animal model to examine the underlying steps of allergy development, focusing on the α-Gal allergy.

The α-Gal glycosylation (galactose-α1,3-galactose; α1,3-Gal; α-Gal) is present on mammalian glycolipids and glycoproteins, except in primates and old-world monkeys. In humans, the gene *GGTA1*, which encodes the enzyme α1,3-galactosyltransferase being responsible for the α-Gal glycosylation, is inactivated. Humans are routinely exposed to α-Gal via food uptake, e.g., dairy products or red meat (2, 3), or via α-Gal-producing bacteria in the digestive tract (4, 5). Due to the missing central tolerance, this provokes an immune reaction to the antigen and α-Gal-specific IgM and IgG equates to 1% of all circulating antibodies in humans. However, an oral tolerance to α-Gal is maintained, unless sensitization occurs. A limited number of sensitized individuals then develop the α-Gal syndrome (AGS) or red meat allergy (6, 7).

The development of the α-Gal syndrome can be divided into two major phases—sensitization and re-exposure to the allergen. Sensitization to α-Gal in humans causes local inflammation and systemic allergy onset by IgE antibody production. Epidemiologic correlations could clearly link α-Gal-specific IgE levels of patients to bites of various tick species in the USA, Europe, Australia, Japan, and Brazil (8–11). Alpha-Gal was shown to be present in the saliva, salivary glands, hemolymph, and gastrointestinal tract of ticks (12–14). Subsequent re-exposure to the α-Gal antigen after the consumption of red meat or mammalian-derived products provokes Urticaria or respiratory symptoms and/or mild to severe gastrointestinal symptoms within 2–6 h (15). Moreover, immediate life-threatening anaphylactic responses to intravenous injections of pharmaceuticals carrying α-Gal glycosylations such as the chimeric monoclonal antibody Cetuximab (16, 17) have been reported (18).

During sensitization to protein antigens, antigen-presenting cells (APCs) such as dendritic cells (DCs) process and present the antigen to naive T-helper cells (Th cells), which differentiate into type 2 T helper (Th2) cells, driven by the master type 2 cytokine IL-4 (19). Th2 cells together with T-follicular helper cells induce a humoral immune response by causing immunoglobulin class-switching of B cells to secrete IgE antibodies (20). IgE antibodies are subsequently bound by high-affinity IgE receptors (FcϵRI) (21–23), present at the surface of mast cells and basophils, which contain inflammatory mediators such as histamine, cytokines, and leukotrienes (24). After re-exposure of a previously sensitized individual to the allergen, the antigen is recognized by the bound IgE antibodies leading to cross-linking of FcϵRI receptors. This then leads to an activation of an intracellular signaling cascade, which results in degranulation and the release of mediators such as histamine and mast cell protease 1 (MCP-1), triggering the allergic response (23). This basic concept was established for protein antigens, if it also applies to a type 2 response to carbohydrates, and what decisive role the cutaneous immune system plays in this multistep process is largely unknown. To understand and ultimately to prevent the development of red meat allergy, predictive animal models are necessary that best reflect the pathophysiology in humans. Mice have been an essential model to examine the function of the host’s immune system after tick bites (25) and to assess the role of immune cells and cytokines, such as IL-4, during allergy development (26). However, mice differ to humans in aspects important to allergy research, such as the structure of the skin and the diet (27). Pigs are more similar to humans with regard to physiology, anatomy, and nutrition, and they too can be genetically modified (28), i.e., to adjust cellular glycosylation patterns (29). Importantly, the structure of the porcine skin is comparable to the human skin, e.g., the thickness of the stratum superficiale dermidis, the average thickness of collagen fibre bundles, the density of the subepidermal capillaries, and the overall microvasculature (30). Consequently, porcine skin has become a substitute for human skin for a variety of applications such as assessing biophysical parameters, diffusivity, permeability, stratum corneum barrier functions, transepidermal water loss, and dermal absorptions (30–32). As not only AGS but also other food allergies require sensitization via the compartment skin (33–36), a porcine allergy model will have wider applications.

In order to establish a porcine AGS model, α-Gal knockout pigs were sensitized by intracutaneous α-Gal injections, mimicking the tick bites. Th2-induced immune responses, and Ig subtype switching were monitored. Finally, to confirm the model, anaphylaxis was induced.

## Materials and methods

### Sensitization of pigs

**Figure 1:**
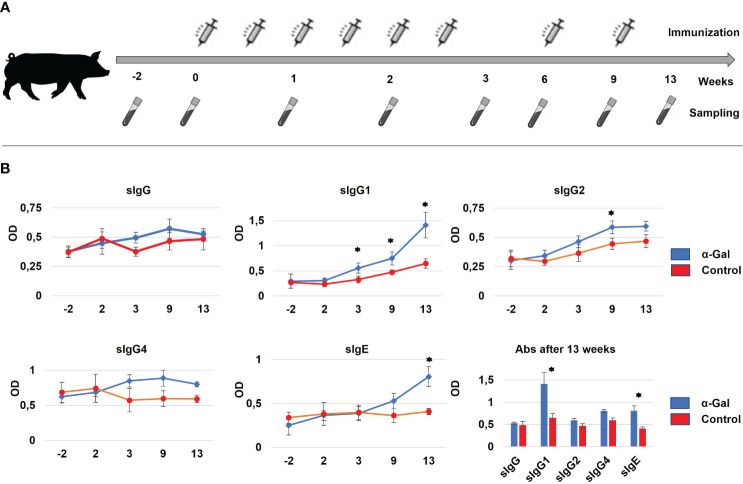
**(A)** Immunization strategy for percutaneous sensitization of pigs. In total, eight immunizations with α-Gal were performed over 9 weeks at days 0, 3, 7, 10, 14, 17, 42, and 63. For each immunization, α-Gal and BSA were 1:2 diluted with Alum to a total volume of 500 µL and applied via 10 intracutaneous injections to an area of 5 cm × 5 cm in the neck. Sampling was performed starting 2 weeks prior to the first immunization until 4 weeks after the last immunization. **(B)** Formation of α-Gal-specific IgG (sIgG) antibodies, IgG1, IgG2, and IgG4 subtypes (sIgG1, sIgG2, and sIgG4) and α-Gal-specific IgE antibodies (sIgE) as determined by ELISA. Significant changes in antibody production between the α-Gal (blue) and control group (red) could be detected after 13 weeks of immunization for sIgG1 and sIgE. N(α-Gal) = 4. N(Control) = 3.

Gene-edited GGTA1 knockout hybrid pigs (37) (German Landrace and minipig cross, adult weight of 80–120 kg) obtained intracutaneous injections of either 25 µg α-Gal BSA (NGP0203, Dextra Laboratories, UK) or 25 µg BSA only (Probumin, vaccine-grade bovine serum albumin, Millipore, Germany), four and three pigs, respectively. Both α-Gal and BSA were 1:2 diluted with Imject®Alum (Thermo Fisher, Germany) to a total volume of 500 µL and applied via 10 intracutaneous injections to an area of 5 cm × 5 cm in the neck. The location was chosen to avoid scratching, which could result in unintended inflammatory reaction. This was repeated twice a week for 3 weeks starting at 6 weeks of age (2 weeks after weaning) followed by two injections at 6 weeks and 9 weeks (**Figure 1**).

During the sensitization studies, pigs were fed commercially available processed animal feed for pigs. Until week 4 of the sensitization protocol, this was piglet feed (Feed 1), afterwards adult feed (Feed 2). Both animal feeds were shown to be low in α-Gal (**Supplementary Figure S1**), to prevent possible immunization of the control animals via the feed. During the immunizations, the core temperature was assessed with a digital thermometer, and skin inflammations at the site of injections were recorded. Blood samples were collected, starting 14 days prior to the first immunization and subsequent at day 0, 7, 14, 21, 42, 56, 63, and 84 (**Figure 1**).

### Intravenous challenge

Four GGTA1 KO pigs, two previously sensitized with α-Gal BSA and two injected with BSA as negative control, were challenged by intravenous (i.v.) injection of 100 mg α-Gal BSA, in 200 µL PBS, 24 weeks after the first sensitization. Serum was collected during the challenge experiment at five time points (0 min, 15 min, 30 min, 45 min, and 60 min) for quantification of porcine mast cell protease (MCP‐1) and histamine levels. Blood pressures and heartbeat rates were measured in 5-min intervals over 60 min.

### Protein isolation

Protein isolation of porcine feed, unprocessed grain, carrageenan, and animal tissues was performed using the Plant Total Protein Extraction Kit (Sigma-Aldrich, Germany, suitable for protein extraction from both animal and plant tissues) according to the manufacturer’s instructions. WT pig tissue was either used untreated, pressure cooked for 10 min, or fried with vegetable oil for 10 min. The tissue was further processed to remove excess oil and moisture and to enable subsequent protein extraction. A total of 200 mg of the samples was ground to a fine powder in liquid nitrogen. Protein concentrations were determined using Advanced Protein Assay Reagent (Sigma-Aldrich, Germany) according to the manufacturer’s instructions. Each sample was aliquoted into three wells of a 96-well plate, and the absorbance was measured in triplicates at 595 nm. The OD values were converted to mg/mL of α-Gal, based on an α-Gal BSA standard curve (NGP0203, Dextra Laboratories, UK) ranging from 0.01 to 20 µg/mL.

### Alpha-Gal ELISA

The 96-well plates were coated with 50 µL of isolated protein solution from feed or porcine tissues and incubated overnight at 4°C. Plates were washed with PBS and blocked with 1% BSA in PBS for 1 h at RT, followed by incubation for 1.5 h at RT with a biotinylated primary anti-alpha-gal monoclonal IgM antibody (Clone M86, Enzo Life Sciences, Germany), diluted 1:50 in the blocking buffer. Biotinylation of the M86 antibody was carried out according to the manufacturer’s instructions for the Abcam Lightning-Link Rapid Type A Biotin Antibody Labeling kit (ab201795). Staining was performed using the ABC reagent kit (Vectastain Elite ABC HRP Kit, Vectorlabs, USA) according to the manufacturer’s instructions. Samples and the standard curve were measured in triplicates. The OD values were converted to mg/mL of α-Gal, based on an α-Gal BSA standard curve (NGP0203, Dextra Laboratories, UK) ranging from 0.01 to 20 µg/mL. The standard deviation was calculated using Excel and indicated as error bars in the respective graph.

### Indirect ELISA to measure alpha-Gal-specific antibodies

To measure the level of antibodies in serum, 96-well Nunc MediSorp ELISA plates (Thermo Fisher, Germany) were coated with 50 µL of 1 µg/mL α-gal epitopes (Galα1-3Galβ1-4GlcNAc-BSA, Dextra, UK) and incubated overnight at 4°C. Each well was washed four times with 200 µL PBS, incubated for 5 min, and subsequently blocked with 100 µL 1% BSA-PBS for 1 h at 37°C. Serum was serially diluted in PBS, and 100 µL was added per well and incubated for 1 h at 37°C. Afterwards, each well was washed four times with PBS. A total of 100 µL of the diluted primary antibody (see **Supplementary Table S1**) was added to the wells, incubated for 1 h at 37°C, followed by the addition of 100 µL of biotinylated secondary antibody (**Supplementary Table S1**), and again incubated at 37°C for 1 h. The plate was washed four times with PBS, and 100 µL of VECTASTAIN ABC Reagent (Biotin-Avidin-Peroxidase system, Vector Laboratories, USA) was added. Staining was performed according to the manufacturer’s instructions. The absorbance at 450 nm was measured with the FLUOstar Omega photometer (BMG Labtech, Germany). The measured OD_450_ value was normalized to the PBS control and the standard curves. The determination of the most suitable serum concentration for the detection of sIgE was based on the standard curve, the correlation factor R^2^, and on an OD_450_ value between 1 and 2. The measured OD_450_ values were compared between the α-Gal and BSA groups and the different time points using an ordinary two-way ANOVA Tukey’s multiple comparisons test conducted on GraphPad Prism.

### Direct ELISA to measure total antibodies

The 96-well Nunc MediSorp ELISA plates (Thermo Fisher, Germany) were coated with 50 µL of serum serially diluted in PBS and incubated overnight at 4°C. Each well was washed four times with PBS and blocked with 100 µL 1% BSA-PBS for 1 h at 37°C. Primary antibodies were diluted with 1% BSA-PBS (**Supplementary Table S1**), and 100 µL was added to the wells for 1 h at 37°C, followed by 100 µL of biotinylated secondary antibody diluted in 1% BSA-PBS (**Supplementary Table S1**) and incubated for a further 1 h at 37°C. The plate was washed four times, and 100 µL of VECTASTAIN ABC Reagent (Vector Laboratories, USA) was added. Staining was performed according to the manufacturer’s instructions, and the absorbance at 450 nm was measured with the FLUOstar Omega photometer (BMG Labtech, Germany).

### Histamine and MCP-1 ELISAs

Serum levels of porcine mast cell protease-1 (MCP‐1) and histamine were quantified by commercial ELISA kits (MyBioSource, MBS264821 or MCA635GA) with a multiskan EX microplate reader (Thermo Fisher, USA) at 450 nm according to the manufacturer’s instructions.

### Skin histology

Skin samples were fixed in 4% formalin for 24 h and embedded in paraffin. Sections were stained with hematoxylin and eosin (HE) or toluidine blue. For mast cell numbers, the mean of at least 10 high-power fields was determined. Inflammatory immune cells were stained using antibodies listed in **Supplementary Table S2** and counted using NIS-ELEMENTS software for analysis (Nikon X-Cite 120 LED).

### Flow cytometry

Single-cell suspensions of skin cells were prepared by digestion with Collagenase IV (Sigma-Aldrich, Germany) for 30 min. Cells were then filtered through a 40-µm strainer. Single-cell suspensions of PBMCs or skin cells were stained with live/dead fixable viability dye eFluor506 or eFluor780 (Thermo Fisher, Germany) in PBS for 10 min and washed with FACS buffer (PBS, 1% FCS). All centrifugation steps were performed at 700×*g* for 1 min at 4°C. Cells were stained for 20 min with surface/extracellular fluorochrome-conjugated antibodies (**Supplementary Table S2**) according to manufacturer’s instructions and washed twice with FACS buffer. Cells were fixed with FoxP3/Transcription kit (eBioscience, Germany) and washed twice in fixation/permeabilization kit buffer. Fixed cells were stained for 20 min with intracellular fluorochrome-conjugated antibodies (**Supplementary Table S2**) and washed with fixation/permeabilization kit buffer and FACS buffer. FACS analysis was performed with a Cytoflex S (four lasers, 13 colors) flow cytometer (Beckman Coulter, USA). Data were analyzed with FlowJo® Single-Cell Analysis Software v10. Surface, and extracellular markers for several immune cell populations are summarized in **Supplementary Table S3**.

### Real-time PCR analysis

Total RNA was isolated from porcine skin by innuSPEED Tissue RNA Kit (Analytik Jena, Germany) according to the manufacturer’s instructions. mRNA levels were quantified by real-time PCR using Fast SybrGreen MasterMix (Applied Biosystems, USA) and run on an ABI 7500 thermocycler (Applied Biosystems, USA) according to the standard protocol for SYBR Premix. Results were evaluated by the cycle threshold method and normalized to the housekeeping gene glyceraldehyde-3-phosphate dehydrogenase (GAPDH).

### Statistical analysis

Statistical analysis was performed using GraphPad PRISM 7.01 (GraphPad Software). Data are shown as means ± standard errors. The statistical differences between comparison groups were assessed by an unpaired t-test. A *p*-value < 0.05 was considered statistically significant.

## Results

Pigs with inactivation of *GGTA1*, deficient in α-Gal, have previously been generated and characterized (29, 38). To avoid unintended sensitization via an (inflamed) colon due to feed with high of α-Gal contents (39), the piglet and adult feed products were evaluated. For comparison, also non-processed and processed wild-type porcine tissues, unprocessed grain (barley), and carrageenan were included in the analysis. Amounts of α-Gal were calculated using an α-Gal BSA standard curve. Both feeds sources showed very low α-Gal levels (**Supplementary Figure S1**). In contrast to other publications, showing stability of the α-Gal antigen after heat denaturation (2), high temperatures significantly reduced the α-Gal concentration in kidney samples (**Supplementary Figure S1**).

### Repeated intracutaneous exposure to α-Gal results in sensitization

Next, we wanted to assess if intracutaneous application of α-Gal can mimic repeated tick bites in humans and resulted in sensitization. In total, eight injections of α-Gal were administered over a 9-week period (**Figure 1A**). To ensure that sensitization itself does not cause anaphylaxis, core body temperature was monitored at the same time. In mice, a drop of the core body temperature is a common read-out for anaphylaxis (40). No significant drop was detected in the α-Gal immunized pigs compared to the control group. However, this test might not be indicative as—in contrast to mice—large animals such as pigs can very likely compensate for a drop in core temperature.

To monitor successful sensitization, blood samples were regularly collected (**Figure 1A**) to determine total and α-Gal-specific Ig levels by ELISA assays. The primary and secondary antibody combinations, which had been optimized for porcine Ig detection, are outlined in **Supplementary Table S1**. When measuring total Ig levels, significant differences between control and sensitized animals were detected for IgG, IgG4, and IgE levels at time points 9 weeks and 13 weeks (**Supplementary Figure S2**). For α-Gal-specific Ig levels only minor changes were observed for sIgG, sIgG2, and sIgG4, while sIgG1 showed a significant increase already by week 3, which continued until week 13. Importantly, α-Gal-specific sIgE levels were significantly increased after the 13-week period, indicating (**Figure 1B**) successful sensitization of the *GGTA1* knockout pigs.

### α-Gal-sensitized pigs show increased immune cell infiltration of the skin

**Figure 2:**
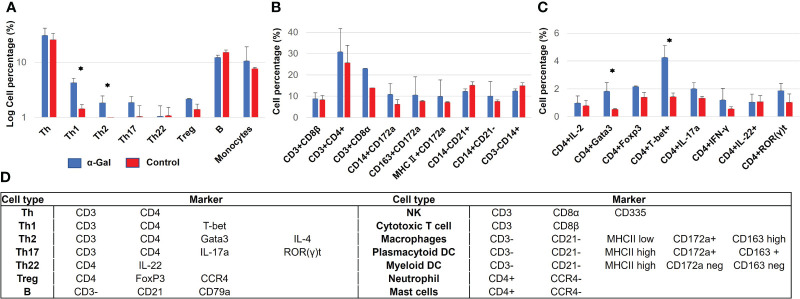
Immune cell populations in blood from α-Gal-immunized and control pigs at week 13 after the first immunization. **(A)** Overview of immune cell populations, showing a significant increase in Th1 and Th2 cells in sensitized pigs. **(B, C)** Detailed analysis of immune cell populations according to several markers which are used to identify several populations at once. **(D)** Markers used for porcine immune cell identification. N(α-Gal)=4. N(Control)=3.

To further clarify the role of immune cells during sensitization and allergy development, isolated PBMCs and skin tissues were analyzed for the presence of Th, Th1, Th2, Th17, Th22, Treg, B, NK, cytotoxic T cells, mast cells, macrophages, dendritic cells, and neutrophils. Alpha-Gal-sensitized pigs had significantly higher numbers of Th1 (CD4^+^, T-bet^+^) and Th2 (CD4^+^, Gata3^+^) cells in the PBMC population compared to the control group (**Figures 2A–C**). Cellular markers for staining and flow cytometry gating are outlined in **Figure 2D**, and antibodies used for detection are shown in **Supplementary Table S2**.

**Figure 3:**
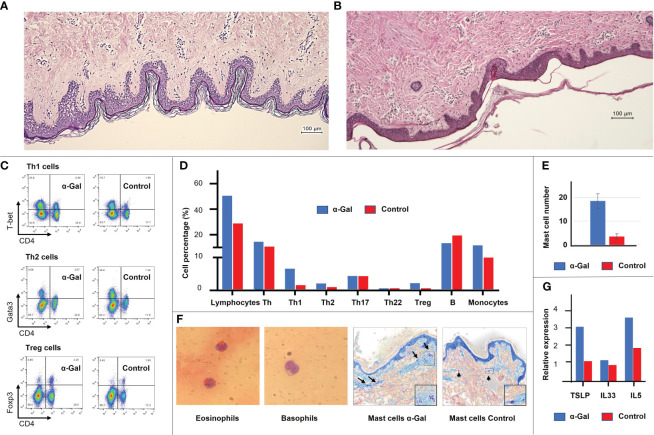
General layers and structure of the human **(A)** and porcine **(B)** skin, revealing high similarities. Immune cell populations and cytokine expression profiles were detected at the site of immunization in the porcine skin. **(C)** Flow cytometric analysis showing an increase in Th1, Th2 and Treg cells in the α-Gal group. **(D)** Detailed immune cell profile of the porcine skin shows an increase in total lymphocyte population, Th1, Th2, Treg cells, and monocytes in the α-Gal group. Shown is the mean of two animals of the α-Gal group compared to two animals of the control group (at least four skin samples per animal). **(E)** Mast cell number in the skin of two α-Gal pigs compared to two control pigs. At least 10 sections per pig were evaluated. **(F)** Giemsa staining of porcine eosinophils and basophils in blood smear. No molecular markers have been characterized yet to identify these cell populations in pigs. Toluidine Blue staining of mast cells. **(G)** Expression levels of type 2 immune alarmins TSLP and IL-33 and the type 2 cytokine IL-5 in the α-Gal and control group.

Four weeks after the last immunization (week 13), one pig from each group was sacrificed. The similar structure of the human and porcine skin was confirmed by histology (**Figures 3A, B**). Immune cell infiltrations of the skin were assessed by flow cytometry. Intracutaneous application of α-Gal led to an increased recruitment of leukocytes, including elevated amounts of Th1, Th2, and Treg cells and monocytes (**Figures 3C, D**). Toluidine Blue staining was used to quantify mast cell numbers. These showed a considerable increase in the skin of sensitized pigs compared to control animals (**Figures 3E, F**, right). So far, no markers for immunohistology have been established to detect porcine eosinophils and basophils. Therefore, Giemsa staining of blood smears was used for detection (**Figure 3F**, left). Since allergic inflammation and skin barrier dysfunction are associated with type 2 immunity, mRNA expression levels of various cytokines and alarmins were measured in skin samples from sensitized and control pigs. Although not significant, due to the limited number of sacrificed animals, increased levels of the type 2 immune alarmins TSLP and IL-33 and the type 2 cytokine IL-5 (**Figure 3G**) were detected in sensitized pigs.

### Re-exposure to α-Gal induces anaphylaxis

**Figure 4:**
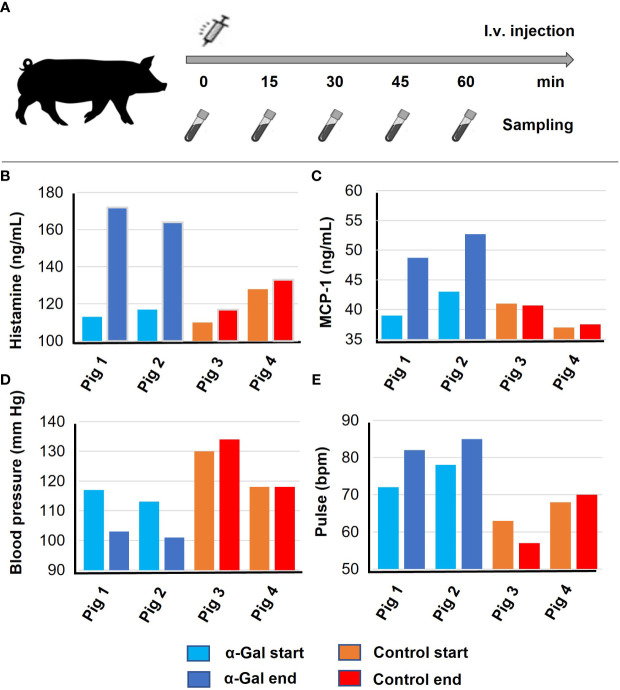
Anaphylaxis after intravenous α-Gal challenge. Results of the first experiments were confirmed and verified based on an independent biological replicate each. Pigs 1 and 2, α-Gal BSA sensitized; Pigs 3 and 4, control pigs (BSA sensitized). **(A)** Timeline for i.v. injection and sample collection. GGTA1-deficient pigs were injected with α-Gal-BSA and blood sampling occurred every 15 min, **(B)** histamine release after 30 min, and **(C)** mast cell protease (MCP-1) levels after 60 min as determined by ELISA. **(D)** Systolic blood pressure and **(E)** pulse were determined 60 min after i.v. injection.

From human patients sensitized to α-Gal, it is known that anaphylaxis can develop following intravenous application of Cetuximab, a monoclonal antibody with α-Gal glycosylations on the Fab fragment (18). We therefore aimed to validate if intravenous exposure to the allergen also elicits anaphylaxis in pre-sensitized pigs. Two α-Gal sensitized and two control pigs were intravenously injected with α-Gal BSA. The experimental setup is outlined in **Figure 4A**. During anaphylaxis, the allergen is recognized by FcϵRI-bound IgE antibodies on mast cells and basophils, activating an intracellular signaling cascade, which results in degranulation and the release of mediators such as histamine and mast cell protease 1 (41). The two α-Gal-sensitized pigs showed a clear increase in histamine and mast cell protease 1 (MCP-1) levels compared to the control pigs (**Figures 4B, C**). As mentioned above, a change in core body temperature was not deemed a reliable anaphylaxis test in pigs. Instead, we monitored the systolic blood pressure and heart rate. Compared to control animals, the α-Gal-sensitized pigs showed a significant drop in blood pressure, which was compensated by an increase in the heartbeat rate (**Figures 4D, E**). These results show for the first time that α-Gal-deficient pigs can be sensitized to α-Gal following intracutaneous exposure and develop anaphylaxis upon re-exposure.

## Discussion

We previously generated α-Gal-deficient pigs through an inactivation of the GGTA1 gene (29, 38). As in humans, these animals produce anti-α-Gal antibodies at steady-state, likely due to α-Gal-expressing gut bacteria. But because of intestinal mucosal tolerance, neither α-Gal-positive microbiota nor α-Gal-rich food leads to adverse immune reactions. As possible for other food allergies such as peanut allergy, sensitization is triggered in the compartment “skin”—for AGS in humans through tick bites.

Here, we show that α-Gal-deficient pigs are a suitable translational model to mimic human α-Gal/red meat allergy. To sensitize animals, a common method is “tape stripping” by which cellophane tape is repeatedly (up to 30 times) applied to the skin and then peeled away to thin the stratum corneum without affecting the underlying epidermis and dermis (42, 43). Subsequently, the sensitizing agent is applied to the same area. To mimic tick bites more closely and to provide a simplified method, we performed the immunization of the pigs by intracutaneous injections instead of tape stripping. While both methods can be performed in the absence of major stress to the animal, e.g., under short-term anesthesia, tape stripping has the disadvantage that it causes local inflammation of a much larger area compared to intracutaneous injections, which may distort the immunological results.

During tick bites, antigens containing α-Gal epitopes are transmitted via the tick saliva, and resident leukocytes, which are interspersed throughout the epidermis and dermis of the skin, are recruited to the bite site (44). Keratinocytes, mast cells, eosinophils, DCs, and macrophages are the first to interact with the mouthparts and the saliva of the tick to promote a local inflammatory response (22, 45). In our model, we used a “synthetic” source for α-Gal, namely, α-Gal-BSA, and alum to mimic the adjuvant effect of tick saliva components such as prostaglandin E2 (PGE2). A shift towards Th2 cell differentiation is induced, e.g., by TSLP, a key epithelial cytokine leading to allergic responses by activating type 2 innate lymphoid cells, basophils, and DCs. TSLP-activated DCs were shown to prime naive T cells to produce the proallergic cytokines (IL-4, IL-5, IL-13, and TNF-α) while downregulating IL-10 and IFN-γ, thus, assuming a role in initiating allergic inflammation (46) by promoting a microenvironment for anti-α-Gal-sIgE production (47, 48) and thus favoring Th2 immune responses (49, 50). This can occur along most barrier surfaces, e.g., the skin, gut, and lungs (51).

In response to the sensitization protocol established here, we could detect an elevated levels of the type 2 immune alarmins TSLP and IL-33 and the type 2 cytokine IL-5. Although not significant, it was sufficient to induce an increase in Th2 cells, Treg cells, mast cells, and monocytes in the skin. Th2 cells further activate eosinophils, promote high antibody levels (52), drive the inflammatory responses, and thus play a significant role in allergy development.

After sensitization, the pigs also showed an increase in Th1 cell populations, both in the skin and blood. The main function of Th1 cells is the production of interferon gamma, IL-2, and tumor necrosis factor to activate macrophages and other immune cells of the cellular immune response (52). Although immune deviation towards Th1 cells was considered for a long time to counterbalance Th2 allergy development, as Th1 cells can antagonize Th2 cellular functions, it could be shown that antigen-specific Th1 cells do not prevent or protect Th2-mediated allergies but even promote additional inflammatory responses (53).

Even though not formally proven for pigs, it can be assumed that the type 2 response finally causes a B-cell isotype switch to the production of IgE antibodies, which requires the type 2 cytokine IL-4 to be initiated. We could detect significantly increased levels of α-Gal-specific sIgE and sIgG1 in our sensitized pigs. In humans, anti α-Gal-sIgE is associated with harmful allergic reactions, and significantly elevated titers of anti-α-Gal sIgG1 antibodies have been observed in AGS patients (54, 55).

One limitation of the porcine model is the current lack of some tools and reagents that are already available for mouse and human studies. For example, in mouse studies, where α-Gal bound to murine serum albumin can be administered, untreated control animals are being used. As no α-Gal bound to porcine serum albumin was available, α-Gal bound to bovine serum albumin (BSA) had to be used to sensitize the pigs, and control animals had to be treated with BSA alone. This may have caused some inflammatory reaction also in the control animals and obscuring the effect of α-Gal in the sensitized animals.

Furthermore, there is no basophil activation test for the pig, which is diagnostic in humans, where it can differentiate between patients prone to an α-Gal allergic reaction from those sensitized with no allergic symptoms (56, 57). This test quantifies basophil degranulation, triggered by a specific antigen, through measurement of activation membrane markers, e.g., CD63 and CD203 (58, 59). Neither marker is so far available for pigs, but the increasing interest in the pig as a biomedical model (60) and for xeno-organ transplantation (61) may soon help to fill such gaps.

The pig is fast becoming an indispensable translational model for biomedical research, including allergy research. The results presented here show the generation of the first translational porcine model for AGS. It enables dietary studies in a physiologically relevant animal model. Both diet and the microbiome play an important role in AGS. Pigs can be fed a human diet, and a comprehensive pig gut microbiome gene reference table has already been established (62). A total of 96% of the functional pathways found in the human listings are present in the pig, and the pig and human catalogues share five times as many genes as the mouse and human catalogues (63). Finally, to understand how the barrier functions can be disrupted is of upmost importance, and allergy research will benefit from the physiological similarity between porcine and human skin and the fact that the porcine immune system is more similar to human than that of mice (64). Pigs are clearly of relevance for food allergy research, and this is now complemented by a new genetically engineered AGS model.

## Data availability statement

The raw data supporting the conclusions of this article will be made available by the authors, without undue reservation.

## Ethics statement

The animal study was reviewed and approved by the Government of Upper Bavaria and performed according to the German Animal Welfare Act and European Union Normative for Care and Use of Experimental Animals. Written informed consent was obtained from the owners for the participation of their animals in this study. The use of human skin samples was approved by ethical vote number 531/17S.

## Author contributions

YW: Conceptualization, Data curation, Formal analysis, Investigation, Methodology, Visualization, Writing – original draft, Writing – review & editing. MH: Conceptualization, Methodology, Writing – original draft, Writing – review & editing. AF: Formal analysis, Visualization, Writing – original draft, Writing – review & editing. FW: Conceptualization, Methodology, Writing – original draft, Writing – review & editing. TB: Conceptualization, Methodology, Validation, Writing – original draft, Writing – review & editing. AS: Conceptualization, Methodology, Resources, Validation, Writing – original draft, Writing – review & editing. KF: Conceptualization, Data curation, Formal analysis, Funding acquisition, Investigation, Methodology, Project administration, Supervision, Validation, Visualization, Writing – original draft, Writing – review & editing.
