# Supplementary material for: Gene-edited pigs: a translational model for human food allergy against alpha-Gal and anaphylaxis
Source: Front Immunol. 2024 Feb 26;15:1358178. doi: 10.3389/fimmu.2024.1358178 (PMC10925645; doi:10.3389/fimmu.2024.1358178)
Supplement: Supplementary file 1 [file DataSheet_1.pdf]

**Supplementary Table 1. Antibodies for ELISA**

| Antibody | Dilution | Conjugate | Catalog |
|---|---|---|---|
| goat anti swine IgG (H&L) | 1:2000 | Biotin | BA-9020 |
| Mouse anti Pig IgG1 | 1:200 | - | MCA635GA |
| Mouse anti Pig IgG2 | 1:2000 | - | MCA636GA |
| Rabbit anti-pig IgG4 | 1:2000 | - | ab232869 |
| Rabbit anti-pig IgE | 1:2000 | - | PAA545Po01 |
| mouse anti-pig IgE | 1:2000 | - | MAA545Po21 |
| mouse-IgGκ BP-B | 1:2000 | Biotin | sc-516142 |
| anti-rabbit IgG | 1:5000 | Biotin | BA-1000 |

**Supplementary Table 2. Antibodies for histology**

| Antibody | Dilution | Conjugate | Subtype | Catalog |
|---|---|---|---|---|
| SLA Class II DR | 1:200 | unlabeled | Mouse/ IgG2b | MA5-28503 |
| Anti-rat IBA1 | 1:2000 | unlabeled | Rabbit/IgG | 019-19741 |
| Anti-human Gata-3 | 1:200 | unlabeled | Rat / IgG2b | 14-9966-80 |
| Anti-Human RORγt | 1:200 | unlabeled | Rat / IgG2a | 14-6988-80 |
| Anti-Human T-bet | 1:200 | unlabeled | Mouse/ IgG1 | 14-5825-80 |
| Anti-human FoxP3 | 1:200 | Biotin | Rat / IgG2a | 13-5773-82 |
| Anti-Porcine CD3e | 1:200 | unlabeled | Mouse/ IgG2b | 4511-01 |
| Anti-Porcine CD4 | 1:200 | unlabeled | Mouse/ IgG2b | 4515-01 |
| Anti-Porcine CD8a | 1:200 | unlabeled | Mouse/ IgG2a | 4520-01 |
| CD117 (c-Kit) | 1:200 | Biotin | Rat / IgG2b | 13-1171-82 |

**Supplementary Table 3. Antibodies for flow cytometry**

| Antibody | Dilution | Conjugate | Subtype | Catalog |
|---|---|---|---|---|
| Anti-human CD21 | 1:20 | BV421 | Mouse/IgG1, κ | 566260 |
| Anti-human CD21 | 1:20 | APC | Mouse/IgG1, κ | 555421 |
| Anti-pig CD3ε | 1:20 | PerCP-Cy5.5 | Mouse/IgG2a, κ | 561478 |
| CD79a | 1:20 | Alexa 647 | Mouse/IgG1 | MCA2538A647T |
| Anti-pig CD14 | 1:20 | FITC | Mouse/IgG2a | MCA1568FT |
| Anti-pig CD4 | 1:20 | FITC | Mouse/IgG2b | MA5-28732 |
| Anti-pig CD8a | 1:20 | Alexa 647 | Mouse/IgG2b, κ | 561475 |

| | | | | |
|---|---|---|---|---|
| Anti-pig CD8b | 1:20 | PE | Mouse/IgG1 | MA5-28441 |
| Anti-pig CD25 | 1:20 | Link700/713 | Mouse/IgG1 | MCA1736GA |
| anti-pig CD172a | 1:100 | PE | Mouse/IgG2b, κ | 561499 |
| anti-human CCR4 | 1-C2 | BV421 | Mouse/IgG1 | 359413 |
| Anti-pig CD45 Monoclonal | 1-C5 | FITC | Mouse/IgG1 | MA5-28383 |
| CD79a | 1-D2 | Alexa 647 | Mouse/IgG1 | MCA2538A647T |
| Langerin Monoclonal Antibody | 1-I7 | APC | Mouse/IgG1 | MA5-23530 |
| Monocytes/Granulocytes Antibody | 1-I8 | PE | Mouse/IgG1 | MA5-28824 |
| SLA Class II DR Monoclonal Antibody | 5-H8 | | Mouse/IgG2b, κ | MA5-28503 |
| CD163 Monoclonal Antibody | 5-I6 | FITC | Mouse/IgG1 | MA5-28292 |
| anti-human T-bet | 1-D3 | eFluor660 | Mouse/IgG1 | 50-5825-82 |
| Anti-Hu IL-4 | 1-I5 | eFluor450 | Mouse/IgG1 | 48-7049-42 |
| Gata-3 Monoclonal Antibody | 4-E8 | PE | rat/IgG2b, κ | 12-9966-42 |
| FOXP3 Monoclonal Antibody | 4-E9 | PE | rat/IgG2a, κ | 12-5773-82 |
| IFN gamma Monoclonal Antibody | 4-F3 | PE | Mouse/IgG1 | 12-7319-42 |
| IL-2 Monoclonal Antibody | 4-F5 | APC | rat/IgG2a, κ | 17-7029-82 |
| IL-17A Monoclonal Antibody | 4-F6 | APC-eFluor® 780 | Mouse/IgG1 | 47-7179-42 |

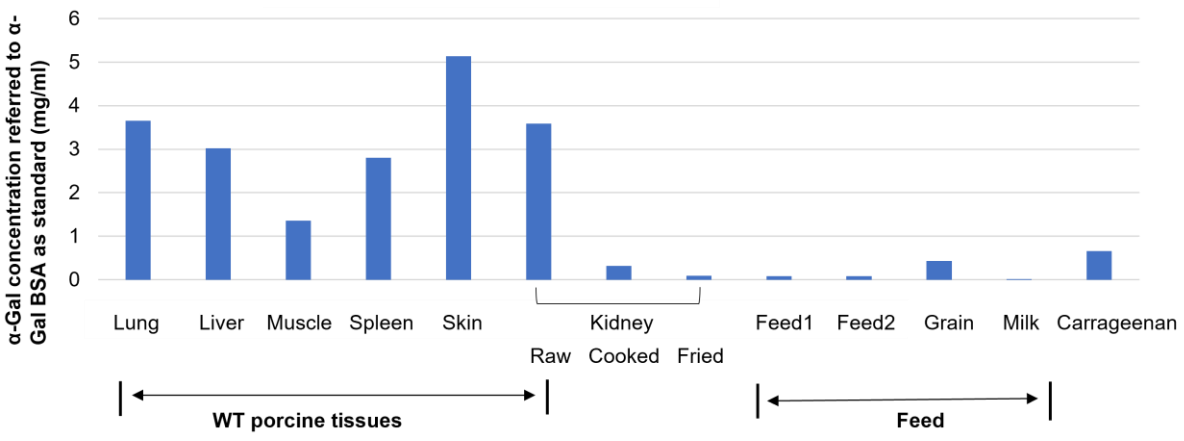


**Supplementary Figure 1**: Concentration of α-Gal in wildtype porcine tissues, processed kidneys and porcine feed. Porcine Feed1 (processed) is for piglets, Feed2 (processed) for adult pigs. We only could detect very low amounts of α-Gal in fried kidneys, Feed1, Feed2 and processed milk. Higher amounts of α-Gal could be detected in unprocessed grain (barley) and carrageenan. Carrageenan, derived from red algae, is a plant-based α-Gal carrying product that differs in the terminal glycosylation structure to which α-Gal is bound. It is frequently used as food additive for human nutrition and linked to cause α-Gal allergy in humans.

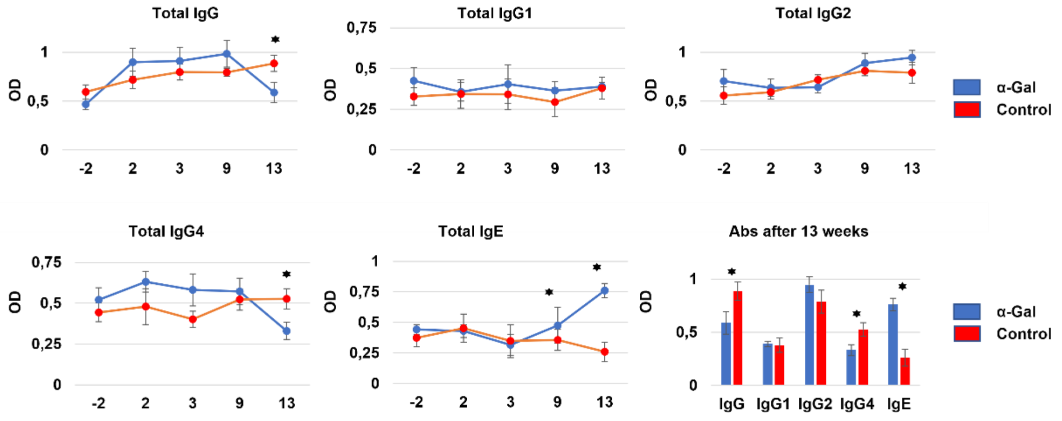


**Supplementary Figure S2**: Total IgG, IgG1, IgG2, IgG4 and IgE antibody levels, starting 2 weeks prior to the first immunization until 13 weeks afterwards. Significant changed levels of total IgG, IgG4 and IgE could be detected after 13 weeks.
